# Relationship between breastfeeding duration, lifestyle and obesity in children aged 3–16 years: a cross-sectional study

**DOI:** 10.3389/fnut.2025.1598141

**Published:** 2025-06-09

**Authors:** Yu Liu, Yiyao Gu, Jie Mu, Zhi Duan, Xixiang Wang, Xiuwen Ren, Lu Liu, Jingjing Xu, Chi Zhang, Shaobo Zhou, Ning Ma, Linhong Yuan, Ying Wang

**Affiliations:** ^1^School of Public Health, Capital Medical University, Beijing Key Laboratory of Environment and Aging, China-British Joint Laboratory of Nutrition Prevention and Control of Chronic Diseases, Beijing, China; ^2^Suzhou Research Center of Medical School, Suzhou Hospital, Affiliated Hospital of Medical School, Nanjing University, Suzhou, China; ^3^School of Biological Sciences, University of Nebraska-Lincoln, Lincoln, NE, United States; ^4^School of Science, Faculty of Engineering and Science, University of Greenwich, Chatham, United Kingdom

**Keywords:** children, obesity, lifestyle, breastfeeding, dietary

## Abstract

**Introduction:**

Childhood obesity is emerging as an increasingly severe public health problem. Effective lifestyle and dietary interventions are urgently needed to prevent childhood obesity. The study explored the association of breastfeeding duration in early life and lifestyle habits with childhood obesity.

**Methods:**

A total of 541 children aged 3–16 at Suzhou Science and Technology City Hospital were included in this analysis. The participants were categorized into obesity group and non-obesity group. Assigned and calculated the score of lifestyle habits and the total score of lifestyle habits and breastfeeding. Logistic regression was used to analyze the risk of obesity with breastfeeding and/or lifestyle habits scores, and ROC curves were applied to evaluate the accuracy of the models. SHapley Additive exPlanation (SHAP) was used to explore the specified impact of variables.

**Results:**

(1) The dietary habits of children with obesity were marked by consuming more meat-based foods, preferring heavier flavors food items, and having a habit of snacking before meals. (2) It is recommended that newborn be breastfed for 4–12 months. (3) Healthy lifestyle habits and prolonged breastfeeding duration are both protective factors for childhood obesity respectively, and the synergistic impact is much more significant.

**Discussion:**

Prolonging breastfeeding duration appropriately and cultivating healthy dietary habits might contribute to prevention of childhood obesity.

## 1 Introduction

Obesity is a major health concern in children and adolescents. The prevalence of obesity among children and adolescents remains high level and an upward trend (1). From 1975 to 2016, the global prevalence of obesity and overweight increased around eight-fold (from 0.7 to 5.6% in girls and from 0.9 to 7.8% in boys) in children aged 5–19 years (2). The estimated global prevalence of overweight and obesity increase from 10 to 20% in boys and from 14 and 24% in girls, hinting that around 400 million children and adolescents may be suffered by 2035 (3). According to the survey, the obesity prevalence rate among preschool children in Jiangsu is 14.0% (4). Alarmingly, the overall prevalence of overweight and obesity among children aged 6–17 years old has soared to 38.60% (5), highlighting a concerning trend in childhood weight management in the region.

The first 1,000 days of life is essential to individual’s development, and would influence the health of adults and aging duration (6). Early life factors contributing to obesity in children include adverse health conditions during pregnancy, unhealthy feeding methods, and environmental risk factor exposures (7). Breastfeeding was considered an effective feeding pattern to prevent obesity and a significant protective factor of obesity (8, 9). Compared with formula-fed children, breastfed children commonly accompanied by a lower incidence of obesity (9). Some studies have found a dose–response protective relationship between the duration of breastfeeding and the risk of overweight/obesity (10, 11). Compared with child accepted breastfeeding for 2 months, those undergone breastfeeding for 5 months showed higher lean mass, skeletal muscle mass and lower total body fat mass. Besides, for the girls, a positive linear relation between breastfeeding duration and trunk lean mass was demonstrated (12).

Sourced from various databases, 28 guidelines, either globally recognized or developed globally by governments, professional organizations, or expert groups, focused on obesity management for children and adolescents with obesity-related comorbidities or severe obesity. They recommended weight loss as the primary treatment method, with only 10 guidelines specifically emphasizing dietary management (13). Childhood is a critical period for the formation of dietary patterns and habits; reasonable guidelines of dietary management are needed for the children with overweight or obesity. Moreover, given the discrepant metabolism status between children and adults, the recommended dietary intervention protocols for adults’ body weight management are not usually suitable for children (14), thus evidence-based specific and efficient clinical body weight management protocol is expected for controlling children obesity. Data from some clinical interventions have demonstrated the impact of change in lifestyle on children obesity or overweight (15). Compared with the control group, 16-week lifestyle intervention (include nutritional counseling and physical exercise) could significantly reduce the BMI *z* score and percentage body fat in obesity child (16).

To date, few studies have explored the linkage of early life diet (represented by breastfeeding duration and dietary habits after weaning) and lifestyle with body weight and obesity, as well as their combined effect on the risk of obesity in child. Therefore, a cross-sectional study was designed to explore the relation between breastfeeding duration and lifestyle with childhood obesity. The data from our study will provide reference for formulating early life feeding method and lifestyle-based preventive and interventional strategy of obesity in children and adolescents.

## 2 Methods

### 2.1 Survey design and study population

From August 26, 2021 to April 30, 2023, children were enrolled at Suzhou Science and Technology City Hospital and Dong Zhu Health Service Center. Inclusion criteria were (i) children aged 3–16 years old enrolled at Suzhou Science and Technology City Hospital and Dong Zhu Health Service Center; (ii) participated in the study after their parents or guardians signed informed consent forms, and the study procedures adhered to the ethical standards and were approved by the relevant ethics committee. Exclusion criteria used in final data analysis were (i) data of breastfeeding time missing; (ii) data of body composition missing; (iii) lack of baseline information for other reasons. A total of 541 subjects, including 320 boys and 221 girls, aged 3–16 years old, were participated in the study. The study protocol was approved by the Committee on Medical Ethics of Suzhou Science and Technology City Hospital (No. IRB202410003RI), and the study procedures followed the ethical standards of the Helsinki Declaration of 1975. Informed consent was signed by all participants or their parents before the investigation.

### 2.2 Anthropometric, hemogram and biochemical measurements

According to the guideline of the World Health Organization (17), the height was measured by a standard height gauge (Seca). Weight-machine was required to calibrated the scale daily (Seca). Body mass index (BMI) was calculated according the formula: BMI (kg/m^2^) = weight (kg)/height (m)/height (m). Children were categorized as obesity and non-obesity according to weight (3–6y) or body mass index (6–16y) [BMI (kg/m^2^)] (details in Supplementary Table S1). Body composition was measured by the Body Composition Analyzer (INBODY S10) (18, 19).

Fasting venous blood (3 mL) was collected by EDTA tubes, and after separating plasma, the samples were stored at −80°C for clinical parameter measurement. Plasma triglyceride (TG) was measured by ILAB600 clinical chemistry analyzer (Instrumentation Laboratory, Lexington, WI, United States). Serum proteins measurement including erythrocyte (10^^12/L^), hemoglobin (g/L), total protein (g/L), albumin (g/L), globulin (g/L) and prealbumin (g/L) were conducted by the clinical laboratory technician. Hemoglobin concentration and blood cell counts in blood samples are measured using the Sysmex XN-1000 (20). Various proteins in the blood are detected by an automatic biochemical analyzer (21).

### 2.3 Lifestyle investigation and score grading

Lifestyle information of the participants was collected according to the questionnaires. Daily dietary habits include exclusive breastfeeding duration (months), energy intake (kcal/d), water intake (ml/d), dietary taste, dietary conditions, food types (per week), and snacks habit. Physical activity level includes weekly frequency and duration.

Lifestyle score (22) was calculated by indexes includes dietary habits (balance dietary habits; imbalance dietary habits including prefer vegetarianism, prefer meat, no staple foods, boredom eating), dietary taste (light flavor, popular flavor, strong flavor), dietary conditions (regular, timed, and quantified meals; irregular conditions including: eat all three meals, but not regularly enough; only eat two meals a day; have supper frequently), water intake (<600 mL/day, ≥600 mL/day), daily food types (<5, 5–12, ≥12), snacks before meals (frequently, occasionally, never), weekly physical activity frequency (never, 1, 2, 3, 4, 5, 6, or 7 times per week) and physical activity duration per time (0, <0.5 h, 0.5–1 h, 1–2 h, 2–3 h, >3 h). Total score including lifestyle score and exclusive breastfeeding duration score (<6 months, 6–24 months, ≥24 months) (23). The scoring grading rule is in Supplementary Table S2.

### 2.4 Statistical analysis

SPSS 26.0 and R 4.2.2 were used for statistical analysis. Measurement data was expressed as mean ± SD or *n* (%). If the data conform to the normal distribution, the t-test was used to compare the differences between groups; otherwise, the rank sum test was applied to compare the difference between groups. The categorical data were expressed as numbers and proportions, and *χ*^2^ test was applied to compare the difference between groups. Pearson or Spearman analysis used for correlation analysis. Logistic regression was run to test the association between the breastfeeding duration, lifestyle and the risk of childhood obesity.

## 3 Results

### 3.1 Demographic characteristics of the participants

As shown in Table 1, the average age of the obesity children group was lower than the non-obesity group (*p* < 0.05). The proportion of boys with obesity was higher than girls (*p* < 0.05). The obesity group children showed higher bodyweight, waist circumference (WC) and BMI than the normal-weight children (*p* < 0.05). Children with obesity showed increased blood erythrocyte, hemoglobin, total protein, albumin, globulin, prealbumin and triglyceride levels as comparing with normal children (*p* < 0.05).

**Table 1:** Demographic characteristics and clinical biochemical indicators of the participants.

Indexes	Obesity (*n =* 233)	Non-obesity (*n =* 308)	*p*-value
Age (years)	8.06 ± 2.76	8.76 ± 2.58	0.003
Gender, *n* (%)			0.020
Female	82 (35.2)	139 (45.1)	
Male	151 (64.8)	169 (54.9)	
Weight (kg)	40.45 ± 14.93	35.9 ± 14.45	<0.001
WC (cm)	68.01 ± 12.60	58.79 ± 6.48	<0.001
BMI (kg/m^2^)	23.39 ± 3.18	15.83 ± 3.18	<0.001
Erythrocyte (10^^12^/L)	4.89 ± 0.31	4.66 ± 0.44	<0.001
Hemoglobin (g/L)	134.23 ± 8.67	131.03 ± 9.56	0.001
Total protein (g/L)	72.02 ± 5.19	69.87 ± 3.60	<0.001
Albumin (g/L)	46.28 ± 2.45	44.63 ± 2.40	0.020
Globulin (g/L)	26.35 ± 3.24	25.24 ± 2.50	0.003
Prealbumin (g/L)	224.23 ± 36.11	215.26 ± 34.54	0.015
Triglyceride (mmol/L)	1.14 ± 0.68	0.94 ± 0.34	0.019

The data was represented as mean ± SD or *n* (%). Quantitative data were analyzed by *t*-test; qualitative data were analyzed using *χ*^2^ test. *P* < 0.05 indicates a statistically significant difference. WC, waist circumference; BMI, body mass index.

### 3.2 Difference in breastfeeding duration and lifestyle

As shown in Table 2, the non-obesity group children showed the much longer breastfeeding duration than the obesity group children, although the difference was not significantly significant (*p* > 0.05). The obesity children showed higher daily energy intake than the non-obesity ones. Difference in dietary habit was observed between groups. The percentage of children preferring meat intake in obesity group was higher than that in the non-obesity group (*p* < 0.05); and the percentage of subjects preferring vegetarianism in obesity group as lower than that in the non-obesity group (*p* < 0.05). More children with obesity prefer popular flavor, while non-obesity children choose lighter flavor (*p* < 0.05). Most children consumed more than 5 types of food items per day, and compared with non-obesity children, the percentage of children consumed 5–12 kinds of food items in obesity group was lower, but the percentage of children consumed > 12 kinds of food items was higher (*p* = 0.013). Obesity children intake snacks before meals more frequently than non-obesity group (*p* = 0.030). The frequency of weekly physical activities of obese children were lower than that among non-obese children (*p* < 0.05), but no difference in the duration of each physical activity between groups. Lifestyle score and combine score of lifestyle and breastfeeding duration were lower in the obesity subjects as comparing with the non-obesity subjects (*p* < 0.05).

**Table 2:** Differences in breastfeeding duration and lifestyle in the participants.

Indexes	Obesity (*N =* 233)	Non-obesity (*N =* 308)	*p*-value
Exclusive breastfeeding duration (month)	7.16 ± 4.80	7.63 ± 4.79	0.134
Energy intake (kcal/d)	1638.91 ± 490.19	1407.32 ± 517.40	<0.001
Dietary habits, *n* (%)			<0.001
Dietary balance	95 (40.8)	104 (33.8)	
Prefer vegetarianism	11 (4.7)	39 (12.7)	
Prefer meat	124 (53.2)	86 (27.9)	
No staple foods	1 (0.4)	4 (1.3)	
Boredom eating	2 (0.9)	75 (24.4)	
Dietary taste, *n* (%)			<0.001
Light flavor	16 (6.9)	53 (17.2)	
Popular flavor	160 (68.7)	165 (53.6)	
Strong flavor	57 (24.5)	90 (29.2)	
Food types (types/d), *n* (%)			0.013
<5	18 (7.7)	28 (9.1)	
5–12	172 (73.8)	250 (81.2)	
>12	43 (18.5)	30 (9.7)	
Snacks before meals, *n* (%)			0.030
Frequently	83 (35.6)	78 (25.3)	
Occasionally	120 (51.5)	189 (61.4)	
Never	30 (12.9)	41 (13.3)	
Physical activity duration (h)	0.94 ± 0.22	0.92 ± 0.50	0.338
Physical activity frequency (weekly)	4.56 ± 1.58	5.07 ± 1.51	<0.001
Lifestyle score	9.60 ± 2.55	10.18 ± 2.37	0.013
Lifestyle and exclusive breastfeeding duration score	10.28 ± 2.55	10.95 ± 2.38	0.005

The data was represented as mean ± SD or *n* (%). Quantitative data were analyzed by *t*-test; qualitative data were analyzed using *χ*^2^ test. *P* < 0.05 indicates a statistically significant difference.

### 3.3 Relationship between breastfeeding duration, lifestyle and obesity

**Figure 1:**
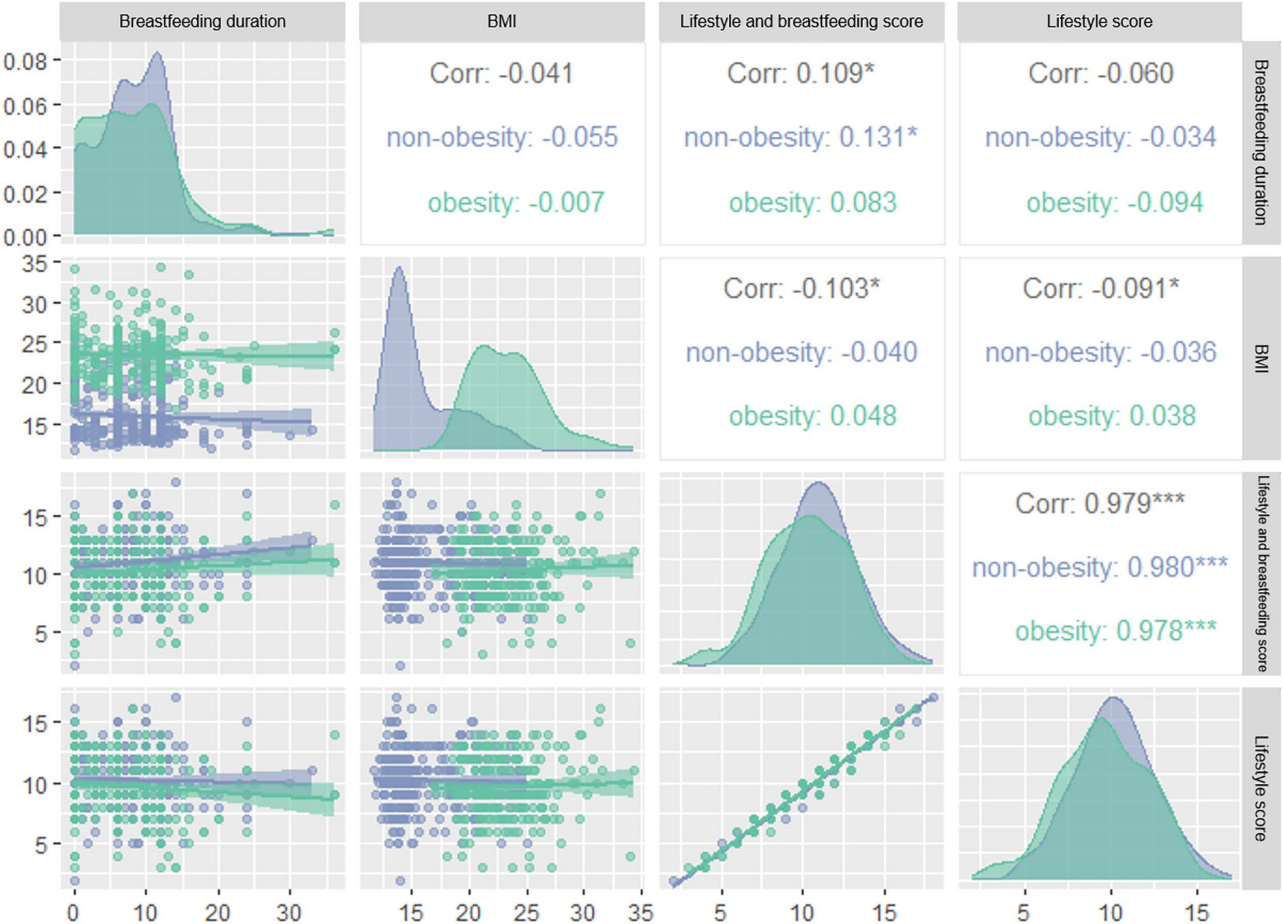
Correlation between breastfeeding duration, BMI, lifestyle score, total score of lifestyle and breastfeeding duration in obesity and non-obesity groups. BMI, body mass index. Blue: non-obesity group, green: obesity group, numbers: correlation coefficients. *p < 0.05, ***p < 0.005.

**Figure 2:**
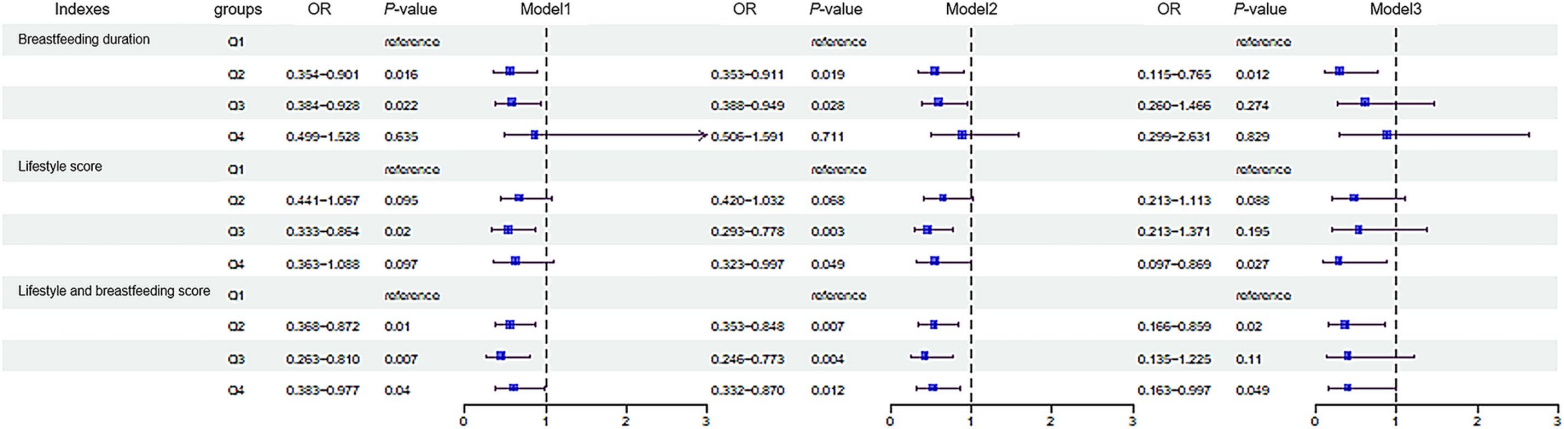
Association of the breastfeeding duration, lifestyle and the risk of childhood obesity. The subjects were divided into Q1 (≤4), Q2 (4–8), Q3 (8–12) and Q4 (≥12) groups according to the breastfeeding duration. Lifestyle score was divided into Q1 (≤8), Q2 (8–10), Q3 (10–12) and Q4 (≥12). Combine score of lifestyle and breastfeeding was divided into Q1 (≤9), Q2 (9–11), Q3 (11–12) and Q4 (≥12). In Model 1, no confounding factors were adjusted; In Model 2, confounding factors including age and gender were adjusted; In Model 3, based on model 2, confounding factors including weight and waist circumference were adjusted. *p* < 0.05 indicates a statistically significant difference. Q, quartile.

As shown in Figure 1, lifestyle score and combine score with breastfeeding duration were negatively correlated with BMI (*r_lifestyle_* = −0.091, *r_combine score_* = −0.103, *p* < 0.05). After dividing the subjects according to quartiles of breastfeeding duration into Q1 (≤4), Q2 (4–8), Q3 (8–12) and Q4 (≥12) groups, we found that the risk of obesity in Q2 group was 0.297 times of that in Q1 group in logistic regression model 3 (Figure 2). We further categorized the subjects according to the quartiles of lifestyle score into Q1 (≤8), Q2 (8–10), Q3 (10–12) and Q4 (≥12) groups, we observed that the risk of obesity in Q3 group was 0.536 times of that in Q1 group in model 1. In model 2, after adjusting the confounder including age and gender, subjects with Q3 and Q4 level of score displayed decreased risk of obesity as comparing with subjects with Q1 level of score. In model 3, we further adjusted bodyweight and WC, subjects in Q4 group consistently showed a decreased risk of obesity as comparing with other groups (*p* < 0.05) (Figure 2). After dividing the subjects according to the quartiles of combine score of lifestyle and breastfeeding duration into Q1 (≤9), Q2 (9–11), Q3 (11–12) and Q4 (≥12) groups, the risk of obesity in Q1, Q2 and Q3 groups decreased significantly in comparison with Q1 group in model 1. In model 2, the association consistently existed after adjusting the confounder including age and gender. We further adjusted the bodyweight and WC in model 3, subjects in Q2 and Q4 group constantly showed a decreased risk of obesity as comparing with Q1 group (Figure 2).

**Figure 3:**
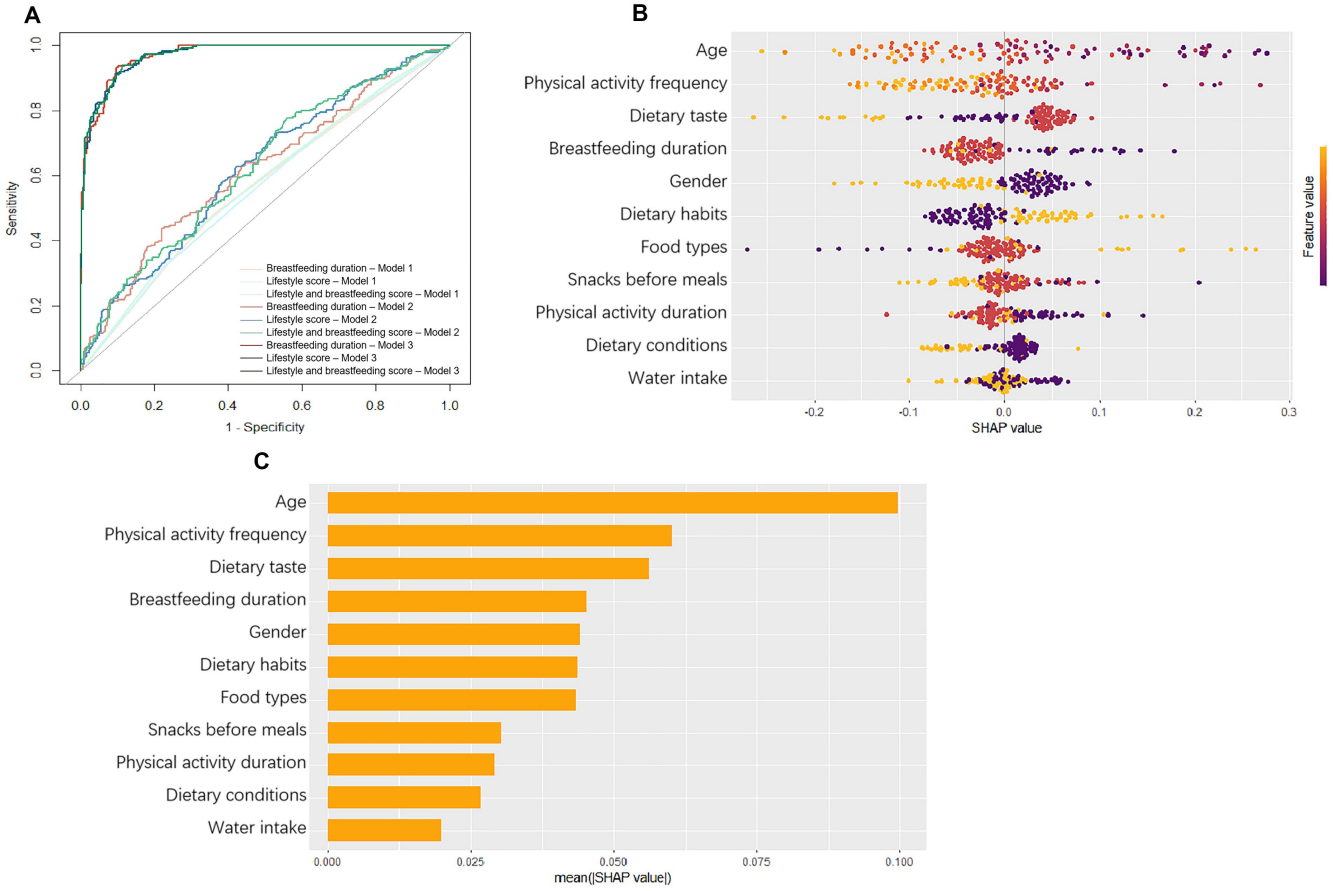
**(A)** ROC curves of the models. In Model 1, no confounding factors were adjusted; In Model 2, confounding factors, including age and gender, were adjusted; In Model 3, based on model 2, confounding factors, including bodyweight and waist circumference, were adjusted. **(B)** The SHapley Additive exPlanation (SHAP) summary plot. **(C)** Influencing factors contribution ranking. The horizontal location means the effect of value on the prediction and the color means the effect of variable on observation.

As shown in Figure 3A and Supplementary Table S3, the results of ROC curves showed that the combine score of lifestyle and breastfeeding duration in model 3 was the best indicator to predict obesity (AUC = 0.978, 95%CI: 0.960–0.982). We further applied the SHapley Additive exPlanation (SHAP) to explore the specified impact of variables on obesity in the compounds of lifestyle and breastfeeding duration score. The SHAP plot showed that age, physical activity frequency, dietary taste and breastfeeding duration were the most important compounds of the model, which negatively contributed to the model (Figures 3B,C).

## 4 Discussion

In this cross-sectional study, we found that early breastfeeding and health dietary habits during childhood are protective factors for childhood obesity.

Multiple studies have shown that, in comparison with artificial feeding, breastfeeding is an efficient feeding method for preventing childhood obesity (24, 25). However, some researchers reported that there was no significant correlation between breastfeeding and obesity (26). In our study, we observed that children who received 4–12 months breastfeeding displayed a significantly lower risk of obesity comparing with those who accepted breastfeeding for less than 4 months, although there was no significant difference in the duration of breastfeeding between children with and without obesity. The conclusions regarding the impact of prolonged breastfeeding duration on childhood obesity or overweight were controversial. Data from Liese’s study found that breastfeeding for more than 1 year significantly reduced the risk of childhood obesity in a dose–response relationship (12). In contrast, others’ results demonstrated that, comparing with the children underwent breastfeeding for 6–9 months, the children accept breastfeeding more than 2 years have decreased risk of obesity and overweight by about 45% (49), but not difference on the risk of obesity as comparing with the child received 1–2 years breastfeeding (11). The World Health Organization recommended the gradual addition of complementary foods to 6-month-old children (27), which could be a potential modifier on the relation between breastfeeding and obesity in children. Therefore, it is necessary to take the addition of complementary foods into the consideration when analyzing the impact of breastfeeding duration on chronic diseases in child.

With the rapid economic development and urbanization progress in China, the incidence of stunted growth and thinness among Chinese children has decreased dramatically (28). Meanwhile, childhood obesity has become a priority public health concern in China. Study has indicated that westernized diet and the change of dietary pattern were major reasons for increased childhood obesity prevalence (29). The living environment of children, such as family and school, is another environmental risk factor that affects childhood obesity (30, 31). For individuals, excessive nutrient intake or unhealthy lifestyle and dietary habits are the most direct risk factors for childhood obesity (32). In this study, we found differences in dietary habits between children with and without obesity, such as preference for meat-based foods, heavy or popular flavor, and habit of snacking before meals.

The relationship between meat intake and obesity is controversial. In a study of Iranian children, the intakes of white meat and poultry were associated with increased risk of general obesity, while the consumption of processed meat was associated with central obesity (33). In a study conducted in China, researchers found a positive association between red meat consumption and overweight and obesity in girls (34). For children and adolescents, the proportion of overweight and obese without weekly meat intake was higher than the proportion of children with daily meat intake (12.3% vs. 11.1%) (35). Data from prospective research confirmed the preventive effect of plant-derived diet on obesity, and the transition from animal-derived foods to plant-derived foods was also recommended to prevent chronic diseases and promote health (36). Patients with obesity commonly prefer “salt, umami, and fatty” foods (37). Consistently, in our study, the children with obesity also self-reported a preference for heavy flavored foods. Additionally, studies have found that children who are breastfed for longer durations develop a preference for neutral flavored foods with less sweetness and fat (38). These results demonstrated that breastfeeding-based dietary favor might partially explain the preventive role of breastfeeding on childhood obesity. Moreover, nowadays, because of habitual fried snacks intakes, children’s intake of high-fat and salty foods has increased correspondingly (39). Especially, the consumption of snacks before stable meals further increased satiety of the child, resulting in reduced sense of hunger and desire for foods, and therefore disturbing the normal consumption of stable meals (40).

Interestingly, in the current study, we also found that the children with obesity consumed more types of food per day in comparison with the non-obesity ones. The dietary diversity score is an indicator constructed to evaluate individual’s dietary habits based on the types of foods consumed. Researchers have found a negative correlation between obesity and the Dietary Diversity Score (DDS) in children, and an increased DDS suggests a reduced risk of overweight (41). Results from another study also revealed that the dietary diversity was positively correlated with overweight in male Chinese (42), and the same relation was observed in the study conducted in preschool children in the United States (43). Discrepant impacts of plant-derived and animal-derived foods on the risk of obesity may be the reason for explaining the different correlations between dietary diversity and childhood obesity. An in-depth investigation is expected to further explore the relation between plant derived DDS and animal derived DDS with childhood obesity in future research. The dietary management for childhood obesity should focus on controlling total daily energy intake, adjusting food proportion, and choosing appropriate food types, rather than simply limiting food intakes. Replacing foods that potentially increased the risk of obesity, including processed foods, snacks, red meat, and refined grains (44) with more light meat, fish, vegetables, and whole grains, might be a smart way to guarantee food diversity, and effectively preventing childhood obesity.

Evidence has shown that combination of dietary intervention with physical activity or psychology treatment could efficiently control body weight (45). In our study, physical activity was a significant factor contributing to the risk of childhood obesity. Overweight adolescents with less daily physical activity habit are susceptible of becoming obese after grow up in the future (46), so adequate physical activity was commonly recommended to the overweight adolescents to prevent obesity in adulthood. Unfortunately, most primary and middle schools could not implement the sports activity guidelines (47), thus, there is still a long way to go for the schools, communities, clinics, and the government to fulfill the physical activity interventional policies (48), to reduce the incidence of childhood obesity and obesity related chronic diseases.

The major innovation of this study is the comprehensive analysis of early life diets (exclusive breastfeeding) and lifestyles (dietary habits and physical activity) with childhood obesity. However, there are still some limitations. Firstly, this study only investigated the duration of breastfeeding without clarifying whether complementary foods have been added and the time for adding complementary foods was unknown, which may be a potential confounding factor of the study. Dietary habits were influenced by the socioeconomic status of family/parents, but the study lacks investigation. Therefore, a more detailed survey needs to be developed in future research. Secondly, the cross-sectional study design made us fail to analyze the time trajectory changes of obesity in child underwent different breastfeeding duration and lifestyle habits, therefore could not determine the contribution of dietary pattern adjustment and food choices to the incidence of childhood obesity. Moreover, the study did not separately explore how breastfeeding duration and lifestyle factors affect obesity differently in males and females, which requires further analysis in the future. Finally, the small sample size was a major limitation of this study, and large scale prospective or randomized controlled trials are needed to provide more reliable and precise scientific evidence.

## 5 Conclusion

In this study, we found that: (1) the dietary habits of children with obesity manifested as consuming more meat-based foods, preferring heavier flavor, and frequently snacking before meals; (2) breastfeeding for 4–12 months significantly reduced the risk of childhood obesity; (3) healthy lifestyle habits and prolonged breastfeeding duration are both protective factors for childhood obesity respectively, and the synergistic impact of healthy lifestyle habits and prolonged breastfeeding on the prevention of childhood obesity is much more significant.

## Data Availability

The raw data supporting the conclusions of this article will be made available by the authors under reasonable request.

## References

[1] Lister NB Baur LA Felix JF Hill AJ Marcus C Reinehr T. Child and adolescent obesity. Nat Rev Dis Primers. (2023) 9:24. doi: 10.1038/s41572-023-00435-4, PMID: 37202378

[2] NCD Risk Factor Collaboration (NCD-RisC). Worldwide trends in body-mass index, underweight, overweight, and obesity from 1975 to 2016: a pooled analysis of 2416 population-based measurement studies in 128·9 million children, adolescents, and adults. Lancet. (2017) 390:2627–42. doi: 10.1016/S0140-6736(17)32129-3, PMID: 29029897 PMC5735219

[3] Nutter S Eggerichs LA Nagpal TS Ramos Salas X Chin Chea C Saiful S. Changing the global obesity narrative to recognize and reduce weight stigma: a position statement from the world obesity federation. Obes Rev. (2024) 25:e13642. doi: 10.1111/obr.13642, PMID: 37846179 PMC13019846

[4] Zhang Y Zhang X Wang X Wu Z Wang Y Ding H. Prevalence and influencing factors of obesity in preschool children in Suzhou, China. An Sist Sanit Navar. (2025) 48:e1105. doi: 10.23938/ASSN.1105, PMID: 40304416 PMC12121459

[5] Yu J Chen S Yang J Zhang X Xue H Ni X. Childhood and adolescent overweight/obesity prevalence trends in Jiangsu, China, 2017-2021: an age-period-cohort analysis. Public Health Nurs. (2025) 42:754–61. doi: 10.1111/phn.13517, PMID: 39737852

[6] Rito AI Buoncristiano M Spinelli A Salanave B Kunešová M Hejgaard T. Association between characteristics at birth, breastfeeding and obesity in 22 countries: the WHO European childhood obesity surveillance initiative – COSI 2015/2017. Obes Facts. (2019) 12:226–43. doi: 10.1159/000500425, PMID: 31030194 PMC6547266

[7] Woo Baidal JA Locks LM Cheng ER Blake-Lamb TL Perkins ME Taveras EM. Risk factors for childhood obesity in the first 1,000 days: a systematic review. Am J Prev Med. (2016) 50:761–79. doi: 10.1016/j.amepre.2015.11.012, PMID: 26916261

[8] Jebeile H Kelly AS O'Malley G Baur LA. Obesity in children and adolescents: epidemiology, causes, assessment, and management. Lancet Diabetes Endocrinol. (2022) 10:351–65. doi: 10.1016/S2213-8587(22)00047-X, PMID: 35248172 PMC9831747

[9] Lackey KA Fehrenkamp BD Pace RM Williams JE Meehan CL McGuire MA. Breastfeeding beyond 12 months: is there evidence for health impacts? Annu Rev Nutr. (2021) 41:283–308. doi: 10.1146/annurev-nutr-043020-011242, PMID: 34115518

[10] Qiao J Dai LJ Zhang Q Ouyang YQ. A meta-analysis of the association between breastfeeding and early childhood obesity. J Pediatr Nurs. (2020) 53:57–66. doi: 10.1016/j.pedn.2020.04.024, PMID: 32464422

[11] Gewa CA. Childhood overweight and obesity among Kenyan pre-school children: association with maternal and early child nutritional factors. Public Health Nutr. (2010) 13:496–503. doi: 10.1017/S136898000999187X, PMID: 19889248

[12] Liese AD Hirsch T von Mutius E Keil U Leupold W Weiland SK. Inverse association of overweight and breast feeding in 9 to 10-y-old children in Germany. Int J Obes Relat Metab Disord. (2001) 25:1644–50. doi: 10.1038/sj.ijo.0801800, PMID: 11753585

[13] Aljahdali AA Cantoral A Peterson KE Perng W Mercado-García A Téllez-Rojo MM. Breastfeeding duration and Cardiometabolic health during adolescence: a longitudinal analysis. J Pediatr. (2024) 265:113768. doi: 10.1016/j.jpeds.2023.113768, PMID: 37802388

[14] Alman KL Lister NB Garnett SP Gow ML Aldwell K Jebeile H. Dietetic management of obesity and severe obesity in children and adolescents: a scoping review of guidelines. Obes Rev. (2021) 22:e13132. doi: 10.1111/obr.13132, PMID: 32896058

[15] Cardel MI Atkinson MA Taveras EM Holm JC Kelly AS. Obesity treatment among adolescents: a review of current evidence and future directions. JAMA Pediatr. (2020) 174:609–17. doi: 10.1001/jamapediatrics.2020.0085, PMID: 32202626 PMC7483247

[16] Heitkamp M Siegrist M Molnos S Brandmaier S Wahl S Langhof H. Obesity genes and weight loss during lifestyle intervention in children with obesity. JAMA Pediatr. (2021) 175:e205142. doi: 10.1001/jamapediatrics.2020.5142, PMID: 33315090 PMC7737153

[17] Seo YG Lim H Kim Y Ju YS Lee HJ Jang HB. The effect of a multidisciplinary lifestyle intervention on obesity status, body composition, physical fitness, and Cardiometabolic risk markers in children and adolescents with obesity. Nutrients. (2019) 11:137. doi: 10.3390/nu11010137, PMID: 30634657 PMC6356576

[18] Riley L Guthold R Cowan M Savin S Bhatti L Armstrong T. The World Health Organization STEPwise approach to non-communicable disease risk-factor surveillance: methods, challenges, and opportunities. Am J Public Health. (2016) 106:74–8. doi: 10.2105/AJPH.2015.302962, PMID: 26696288 PMC4695948

[19] National Health Commission of the People's Republic of China. Growth standard for children under 7 years of age National Health Commission of the People's Republic of China. China (Beijing). (2022).

[20] National Health Commission of the People's Republic of China (2018). National Health Commission of the People's Republic of China screening for overweight and obesity among school-age children and adolescents. China (Beijing).

[21] Ma J Qiao Y Zhao P Li W Katzmarzyk PT Chaput JP. Breastfeeding and childhood obesity: a 12-country study. Matern Child Nutr. (2020) 16:e12984. doi: 10.1111/mcn.12984, PMID: 32141229 PMC7296809

[22] Mendieta-Gutiérrez C Chávez-González S Rodríguez-Romero BI Sánchez-Garrido JA Figueroa-Gómez A Licona-Vela JB. Establishment of reference intervals for complete blood count in times of COVID-19 and vaccination. Biochem Med Zagreb. (2023) 33:020701. doi: 10.11613/BM.2023.020701, PMID: 37143716 PMC10152613

[23] Chang Q Chen HL Wu NS Gao YM Yu R Zhu WM. Prediction model for severe *Mycoplasma pneumoniae* pneumonia in pediatric patients by admission laboratory indicators. J Trop Pediatr. (2022) 68:59. doi: 10.1093/tropej/fmac059, PMID: 35903920

[24] Rozanski A Sakul S Narula J Berman D. Assessment of lifestyle “vital signs” in healthcare settings. Prog Cardiovasc Dis. (2023) 77:107–18. doi: 10.1016/j.pcad.2023.02.002, PMID: 36848965

[25] Lee PH Macfarlane DJ Lam TH Stewart SM. Validity of the international physical activity questionnaire short form (IPAQ-SF): a systematic review. Int J Behav Nutr Phys Act. (2021) 8:115. doi: 10.1186/1479-5868-8-115 PMC3214824 22018588

[26] Hediger ML Overpeck MD Kuczmarski RJ Ruan WJ. Association between infant breastfeeding and overweight in young children. JAMA. (2001) 285:2453–60. doi: 10.1001/jama.285.19.2453, PMID: 11368697

[27] World Health Organization (2002). Complementary feeding: Report of the global consultation and summary of guiding principles for complementary feeding of the breastfed child. WHO Team on Maternal, Newborn, Child & Adolescent Health & Aging (MCA) and Nutrition and Food Safety (NFS).

[28] Dong Y Lau PWC Dong B Zou Z Yang Y Wen B. Trends in physical fitness, growth, and nutritional status of Chinese children and adolescents: a retrospective analysis of 1·5 million students from six successive national surveys between 1985 and 2014. Lancet Child Adolesc Health. (2019) 3:871–80. doi: 10.1016/S2352-4642(19)30302-5, PMID: 31582349

[29] Wang Y Wang L Xue H Qu W. A review of the growth of the fast food industry in China and its potential impact on obesity. Int J Environ Res Public Health. (2016) 13:1112. doi: 10.3390/ijerph13111112, PMID: 27834887 PMC5129322

[30] Zhou S Cheng Y Cheng L Wang D Li Q Liu Z. Association between convenience stores near schools and obesity among school-aged children in Beijing, China. BMC Public Health. (2020) 20:150. doi: 10.1186/s12889-020-8257-0, PMID: 32005214 PMC6995088

[31] Li M Dibley MJ Yan H. School environment factors were associated with BMI among adolescents in Xi'an city, China. BMC Int Health Hum Rights. (2011) 11:792. doi: 10.1186/1471-2458-11-792 PMC3198712 21988882

[32] Zhu Z Tang Y Zhuang J Liu Y Wu X Cai Y. Physical activity, screen viewing time, and overweight/obesity among Chinese children and adolescents: an update from the 2017 physical activity and fitness in China-the youth study. BMC Public Health. (2019) 19:197. doi: 10.1186/s12889-019-6515-9, PMID: 30767780 PMC6376726

[33] Khodayari S Sadeghi O Safabakhsh M (2022) Mozaffari-Khosravi H Meat consumption and the risk of general and central obesity: the Shahedieh study BMC Res Notes 15,:339. doi: 10.1186/s13104-022-06235-5 36320017 PMC9628015

[34] Jakobsen DD Brader L Bruun JM. Association between Food, Beverages and Overweight/Obesity in Children and Adolescents-A Systematic Review and Meta-Analysis of Observational Studies. Nutrients. (2023) 15:764. doi: 10.3390/nu15030764 PMID: 36771470 PMC9920526

[35] Shin SM. Association of Meat Intake with overweight and obesity among school-aged children and adolescents. J Obes Metab Syndr. (2017) 26:217–26. doi: 10.7570/jomes.2017.26.3.217, PMID: 31089520 PMC6484919

[36] Gibbs J Cappuccio FP. Plant-based dietary patterns for human and planetary health. Nutrients. (2022) 14:1614. doi: 10.3390/nu14081614, PMID: 35458176 PMC9024616

[37] van Langeveld AWB Teo PS de Vries JHM Feskens EJM de Graaf C Mars M. Dietary taste patterns by sex and weight status in the Netherlands. Br J Nutr. (2018) 119:1195–206. doi: 10.1017/S0007114518000715, PMID: 29759103

[38] Nguyen AN van Langeveld AWB de Vries JHM Ikram MA de Graaf C Mars M. Dietary taste patterns in early childhood: the generation R study. Am J Clin Nutr. (2021) 113:63–9. doi: 10.1093/ajcn/nqaa296, PMID: 33184622 PMC7779211

[39] Zou Y Huang L He M Zhao D Su D Zhang R. Sedentary activities and food intake among children and adolescents in the Zhejiang Province of China: a cross-sectional study. Nutrients. (2023) 15:3745. doi: 10.3390/nu15173745, PMID: 37686777 PMC10490322

[40] Papakonstantinou E Orfanakos N Farajian P Kapetanakou AE Makariti IP Grivokostopoulos N. Short-term effects of a low glycemic index carob-containing snack on energy intake, satiety, and glycemic response in normal-weight, healthy adults: results from two randomized trials. Nutrition. (2017) 42:12–9. doi: 10.1016/j.nut.2017.05.011, PMID: 28870473

[41] Tao C Zhao Q Glauben T Ren Y. Does dietary diversity reduce the risk of obesity? Empirical evidence from rural school children in China. Int J Environ Res Public Health. (2020) 17:8122. doi: 10.3390/ijerph17218122, PMID: 33153180 PMC7662578

[42] Tian X Wu M Zang J Zhu Y Wang H. Dietary diversity and adiposity in Chinese men and women: an analysis of four waves of cross-sectional survey data. Eur J Clin Nutr. (2017) 71:506–11. doi: 10.1038/ejcn.2016.212, PMID: 27848939

[43] Fernandez C Kasper NM Miller AL Lumeng JC Peterson KE. Association of Dietary Variety and Diversity with Body Mass Index in US preschool children. Pediatrics. (2016) 137:e20152307. doi: 10.1542/peds.2015-2307, PMID: 26908657 PMC4771127

[44] Liberali R Kupek E Assis MAA. Dietary patterns and childhood obesity risk: a systematic review. Child Obes. (2020) 16:70–85. doi: 10.1089/chi.2019.0059, PMID: 31742427

[45] De Miguel-Etayo P Bueno G Garagorri JM Moreno LA. Interventions for treating obesity in children. World Rev Nutr Diet. (2013) 108:98–106. doi: 10.1159/000351493, PMID: 24029793

[46] Hills AP Andersen LB Byrne NM. Physical activity and obesity in children. Br J Sports Med. (2011) 45:866–70. doi: 10.1136/bjsports-2011-090199, PMID: 21836171

[47] Guthold R Stevens GA Riley LM Bull FC. Global trends in insufficient physical activity among adolescents: a pooled analysis of 298 population-based surveys with 1·6 million participants. Lancet Child Adolesc Health. (2020) 4:23–35. doi: 10.1016/S2352-4642(19)30323-2, PMID: 31761562 PMC6919336

[48] Pate RR Flynn JI Dowda M. Policies for promotion of physical activity and prevention of obesity in adolescence. J Exerc Sci Fit. (2016) 14:47–53. doi: 10.1016/j.jesf.2016.07.003, PMID: 29541118 PMC5801719

[49] Fallahzadeh H Golestan M Rezvanian T Ghasemian Z. Breast-feeding history and overweight in 11 to 13-year-old children in Iran. World J Pediatr. (2009) 5:36–41. doi: 10.1007/s12519-009-0006-5, PMID: 19172330

