# Supplementary material for: Relationship between breastfeeding duration, lifestyle and obesity in children aged 3–16 years: a cross-sectional study
Source: Front Nutr. 2025 Jun 9;12:1598141. doi: 10.3389/fnut.2025.1598141 (PMC12183054; doi:10.3389/fnut.2025.1598141)
Supplement: Supplementary file 1 [file Table_1.docx]

Supplementary Material

# Supplementary Tables

**Table S1-1 Categorized of obesity in children aged 3-6**

(aged 3-6, Kg)

| Age (years) | Male | | | Female | | |
| --- | --- | --- | --- | --- | --- | --- |
|  | +1SD | +2SD | +3SD | +1SD | +2SD | +3SD |
| 3.0- | 16.8 | 18.3 | 20.1 | 16.5 | 18.1 | 19.9 |
| 4.0- | 16.6 | 18.1 | 20.1 | 16.4 | 18.0 | 20.0 |
| 5.0- | 16.7 | 18.4 | 20.7 | 16.4 | 18.2 | 20.5 |

**Table S1-2 Categorized of obesity in children aged 6-18**

(aged 6-18, Kg/m^2^)

| Age (years) | Male | Female | Age (years) | Male | Female |
| --- | --- | --- | --- | --- | --- |
| 6.0- | 17.7 | 17.5 | 12.5- | 24.7 | 24.5 |
| 6.5- | 18.1 | 18.0 | 13.0- | 25.2 | 25.0 |
| 7.0- | 18.7 | 18.5 | 13.5- | 25.7 | 25.6 |
| 7.5- | 19.2 | 19.0 | 14.0- | 26.1 | 25.9 |
| 8.0- | 19.7 | 19.4 | 14.5- | 26.4 | 26.3 |
| 8.5- | 20.3 | 19.9 | 15.0- | 26.6 | 26.6 |
| 9.0- | 20.8 | 20.4 | 15.5- | 26.9 | 26.9 |
| 9.5- | 21.4 | 21.0 | 16.0- | 27.1 | 27.1 |
| 10.0- | 21.9 | 21.5 | 16.5- | 27.4 | 27.4 |
| 10.5- | 22.5 | 22.1 | 17.0- | 27.6 | 27.6 |
| 11.0- | 23.0 | 22.7 | 17.5- | 27.8 | 27.8 |
| 11.5- | 23.6 | 23.3 | 18.0- | 28.0 | 28.0 |
| 12.0- | 24.1 | 23.9 |  |  |  |

**Table S2 Score Grading**

| Indexes | 0 | 1 | 2 | 3 | 4 | 5 | 6 |
| --- | --- | --- | --- | --- | --- | --- | --- |
| Exclusive breastfeeding duration (month) | < 6 | 6-24 | ≥ 24 |  |  |  |  |
| Dietary habits | Imbalance | Balance |  |  |  |  |  |
| Dietary taste | Strong flavor | Popular flavor | Light flavor |  |  |  |  |
| Dietary conditions | Irregularly | Regularly |  |  |  |  |  |
| Water intake (ml/day) | < 600 | 600 |  |  |  |  |  |
| Food types (types/day) | < 5 | 5-12 | ≥ 12 |  |  |  |  |
| Snacks before meals | Frequently | Occasionally | Never |  |  |  |  |
| Physical activity frequency (times/week) | 0, 1 | 2 | 3 | 4 | 5 | 6 | 7 |
| Physical activity duration (hours/time) | 0 | < 0.5 | 0.5-1 | 1-2 | 2-3 | > 3 |  |

**Table S3 Comparison of ROC curves for breastfeeding duration and lifestyle**

| Models | AUC | 95%CI | *P-value* | Sensitivity | Specificity | Youden index | Cut-off |
| --- | --- | --- | --- | --- | --- | --- | --- |
| **Breastfeeding duration (month)** | | | | | | | |
| Model 1 | 0.565 | (0.516,0.614) | 0.009 | 0.481 | 0.640 | 0.120 | 0.433 |
| Model 2 | 0.618 | (0.570,0.666) | < 0.001 | 0.438 | 0.779 | 0.217 | 0.483 |
| Model 3 | 0.971 | (0.959,0.982) | < 0.001 | 0.936 | 0.899 | 0.835 | 0.387 |
| **Lifestyle score** | | | | | | | |
| Model 1 | 0.562 | (0.514,0.611) | 0.012 | 0.322 | 0.773 | 0.095 | 0.470 |
| Model 2 | 0.621 | (0.573,0.668) | < 0.001 | 0.627 | 0.578 | 0.205 | 0.418 |
| Model 3 | 0.970 | (0.959,0.982) | < 0.001 | 0.914 | 0.903 | 0.817 | 0.413 |
| **Lifestyle and breastfeeding score** | | | | | | | |
| Model 1 | 0.574 | (0.526,0.623) | 0.003 | 0.382 | 0.744 | 0.125 | 0.469 |
| Model 2 | 0.627 | (0.580,0.674) | < 0.001 | 0.777 | 0.442 | 0.218 | 0.374 |
| Model 3 | 0.978 | (0.960,0.982) | < 0.001 | 0.940 | 0.886 | 0.826 | 0.354 |

Model 1, no confounding factors were adjusted; Model 2, confounding factors including age and gender were adjusted; Model 3, based on model 2, confounding factors including weight and waist circumference were adjusted. *P* < 0.05 indicates a statistically significant difference.
